# Retinal microvasculature and macular–choroidal thickness in oral contraceptive users: an OCTA and OCT comparative study

**DOI:** 10.1186/s40942-025-00775-1

**Published:** 2025-11-28

**Authors:** Alana Ferreira Gomes Dias, Márcio Fragoso Vieira, Francisco Vagnaldo Fechine Jamacaru, Maria Elisabete Amaral de Moraes

**Affiliations:** 1 Department of Ophthalmology, Faculty of Medicine, INTA University Center, Sobral, CE Brazil; 2 Department of Obstetrics and Gynecology, Faculty of Medicine, Federal University of Ceará, Sobral, CE Brazil; 3 Drug Research and Development Center (NPDM), Faculty of Medicine, Federal University of Ceará, Fortaleza, CE Brazil

**Keywords:** Hormonal oral contraceptives, Retinal vasculature, Choroid, Angiography, Optical coherence tomography

## Abstract

**Background:**

Potential retinal vascular changes associated with hormonal contraceptive use remain insufficiently characterized in the literature. Optical coherence tomography angiography (OCTA) enables a noninvasive and highly detailed evaluation of retinal microvasculature. This study aimed to assess the superficial and deep retinal capillary plexuses, and macular and choroidal thickness in women using combined oral contraceptives (COC) or progestogen-only contraceptives (POC) compared with women who had never used hormonal contraceptives. The potential influence of the duration of use was also investigated.

**Methods:**

A cross-sectional analysis was conducted in 120 healthy women aged 20–40 years, equally distributed into the COC, POC, and control groups. OCTA was used to measure foveal avascular zone (FAZ) parameters and vessel densities of the superficial and deep plexuses in a 3 × 3 mm scan. Macular and choroidal thickness were obtained using spectral-domain optical coherence tomography. One randomly selected eye of each participant was analyzed. Intergroup comparisons were performed using analysis of variance (ANOVA) or Kruskal–Wallis test, with appropriate post-hoc analyses; significance was set at 5%.

**Results:**

No significant differences were observed among the groups in FAZ parameters, macular thickness, or choroidal thickness. A small but statistically significant reduction in vessel density of the nasal parafoveal sector of the superficial capillary plexus was found in the POC subgroup with less than two years of use compared with controls (*P* = 0.033; post-hoc *P* = 0.049). No other significant differences were detected in the superficial or deep plexuses.

**Conclusion:**

Hormonal contraceptive use was not associated with significant alterations in FAZ parameters, macular or choroidal thickness, or overall retinal vessel density. A slight reduction in nasal parafoveal vessel density was observed in women with POC use for less than 2 years compared with controls, although the clinical significance of this finding remains uncertain. These results suggest that retinal vascular changes associated with hormonal contraceptives are minimal, underscoring the need for longitudinal studies with larger samples to confirm these observations.

**Supplementary Information:**

The online version contains supplementary material available at 10.1186/s40942-025-00775-1.

## Background

Since the approval of the first oral contraceptive in the 1960s, this method has remained the most widely used form of family planning. These medications are composed of a combination of estrogen and progestin or progestin alone and are available in various formulations (associations and hormone concentrations) [1].

Combined oral contraceptives (COC) have been associated with several ocular pathologies. However, analyses suggest that the only statistically significant effect is a two-fold increase in the risk of retinal vascular lesions [2]. Previous studies have reported associations between COC use and changes in retinal and choroidal thickness after one year of use, and that long-term users may develop ocular changes. In such cases, optical coherence tomography (OCT) may be a useful follow-up tool [3].

Despite the known association between COC and venous thromboembolism, as well as the increased risk of retinal vascular diseases, only two small-sample studies have evaluated the retinal microvasculature in COC users using OCT angiography (OCTA). Both studies reported differences in vascular parameters among women using a specific COC formulation [4, 5].

Although several studies have evaluated ocular effects of hormonal contraceptives, further research is needed to enhance the application and interpretation of OCTA in retinal vascular diseases, especially in patients using chronic medications, such as women taking hormonal contraceptives.

This study aimed to evaluate the superficial and deep retinal vasculature using OCTA and macular and choroidal thickness using OCT in users of hormonal oral contraceptives. This study also assessed the influence of the duration of contraceptive use on retinal vascular and structural parameters.

## Methods

An observational, cross-sectional, comparative study was conducted involving three groups of women: users of COC, users of progestogen-only oral contraceptives (POC), and women who had never used hormonal contraception. The study was approved by the Research Ethics Committee (CEP) of the Federal University of Ceará - CEP/UFC/PROPESQ (CAAE 55193422.1.0000.5054). All participants provided written informed consent prior to enrollment. This study was conducted at Dr. José Nilson Clinic, Sobral/CE, Brazil, between May 2022 and December 2023.

### Subjects

A total of 120 healthy women aged 20–40 years were enrolled in the study and divided into three groups of 40 each: COC users, POC users, and non-users. All participants had a visual acuity of 20/30 or better and no history of systemic or ocular disease. Eligible eyes had a spherical equivalent between −5.00 D and +5.00 D, and no high-myopic eyes were enrolled. Minor visual acuity reduction was attributable to uncorrected refractive error. The exclusion criteria included chronic systemic conditions (such as hypertension or diabetes), ocular trauma or previous ocular surgery, smoking, drug use, and any other factor that could impair the quality of OCTA images.

### Procedures

All participants underwent a complete ophthalmological examination. OCT and OCTA were performed using the Revo NX system (Optopol, Poland). Examinations were performed at varying times of the day according to participant availability. Macular and choroidal thicknesses were evaluated using standard macular OCT scans in both eyes. Subsequently, the device mode was switched to OCTA to assess the foveal avascular zone (FAZ) and capillary plexus parameters. OCTA scans were acquired using a 3 × 3 mm macular protocol. All macular and vessel-density metrics were automatically generated by the device’s software. Subfoveal choroidal thickness was the only manually obtained parameter and was measured using the caliper tool from the outer border of the retinal pigment epithelium to the chorioscleral junction. Only scans with signal strength ≥ 7/10 were included. Images with motion artifacts, segmentation errors, or defocus blur were excluded and repeated when necessary. All scans were reviewed by a single trained grader to ensure consistency across evaluations. Segmentation errors or motion artifacts prompted repeat imaging when feasible. For quantitative OCTA analysis, the device’s default segmentation boundaries were applied: the superficial capillary plexus (SCP) slab extended from the internal limiting membrane to the inner border of the inner plexiform layer (IPL), and the deep capillary plexus (DCP) slab extended from the outer border of the IPL to the outer plexiform layer. No projection-artifact removal tools were applied during image processing.

**Fig. 1:**
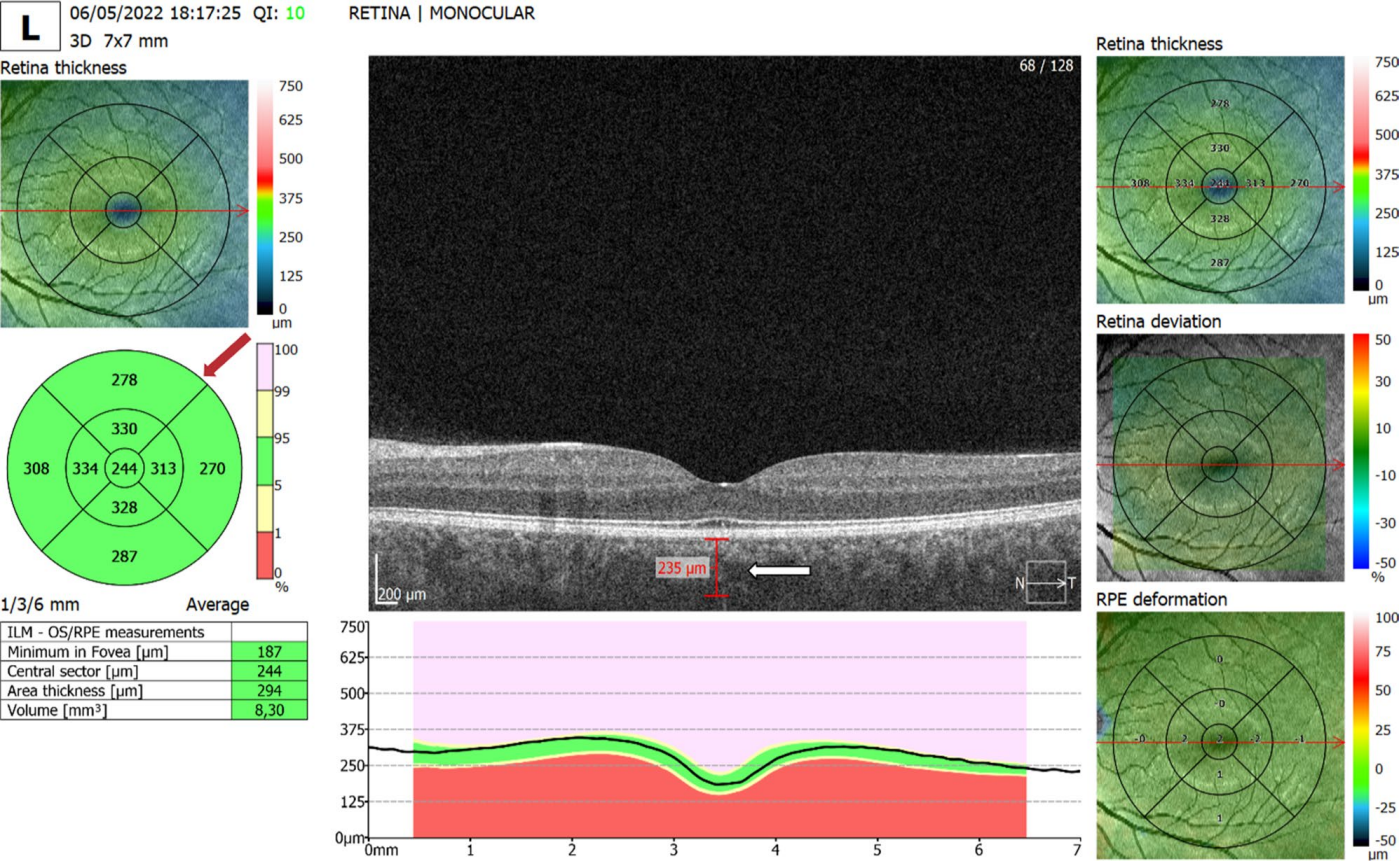
Structural macular OCT with automated thickness maps and subfoveal choroidal measurement. Representative 7 × 7 mm macular OCT showing automated ETDRS thickness maps (red arrow) and manual subfoveal choroidal thickness measurement (white arrow). Scale bar = 200 μm. Signal strength = 10/10

The ophthalmological variables assessed by OCT (expressed in µm) were foveal, parafoveal, and perifoveal thickness (superior, inferior, nasal, and temporal) and subfoveal choroidal thickness (Fig. 1).

**Fig. 2:**
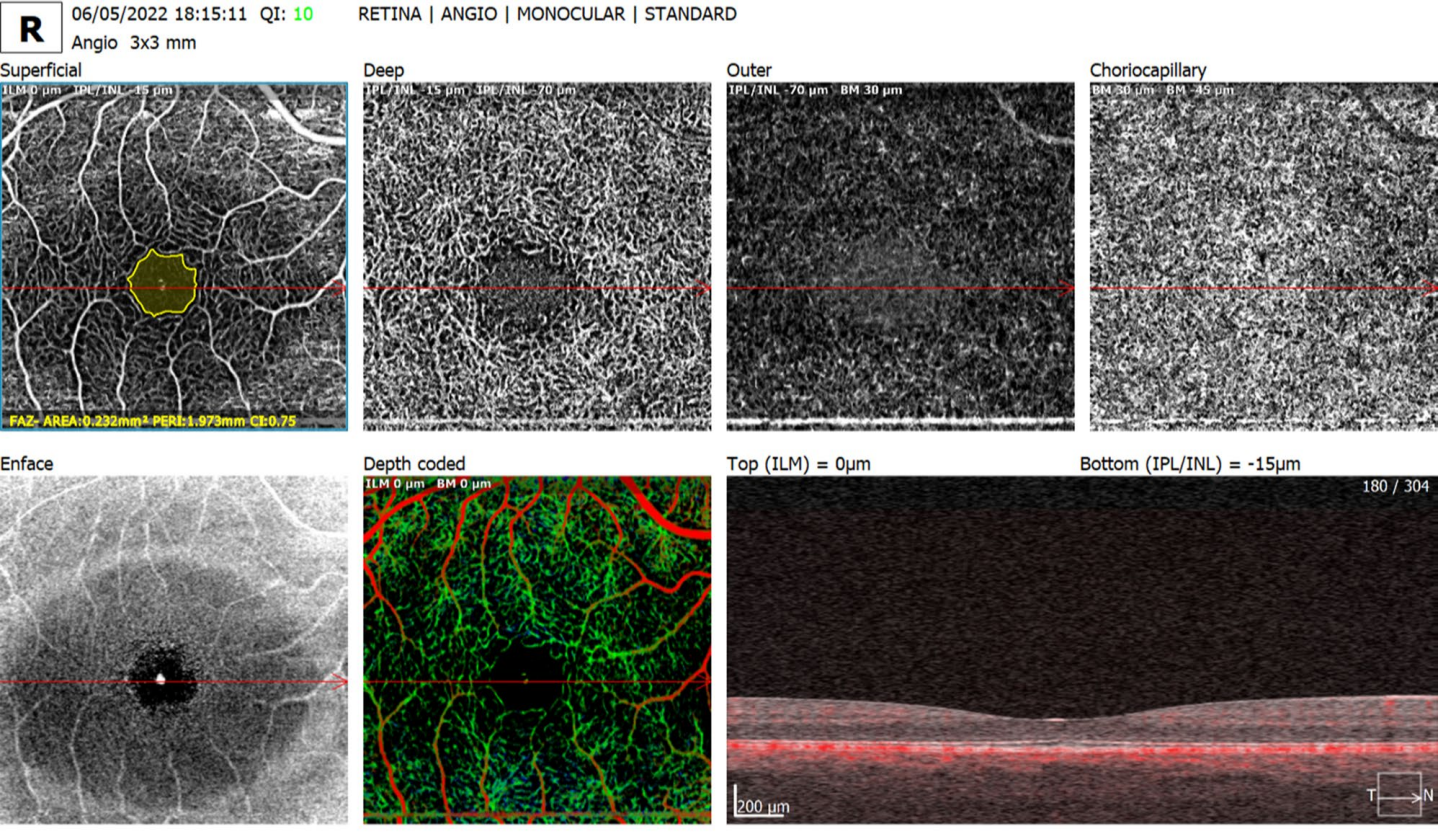
OCTA 3 × 3 mm scan showing FAZ measurement and multilayer vascular slabs. Superficial plexus with FAZ outline, plus deep, outer retina, and choriocapillaris slabs. Default device segmentation was used. Scale bar = 200 μm; signal strength = 10/10

**Fig. 3:**
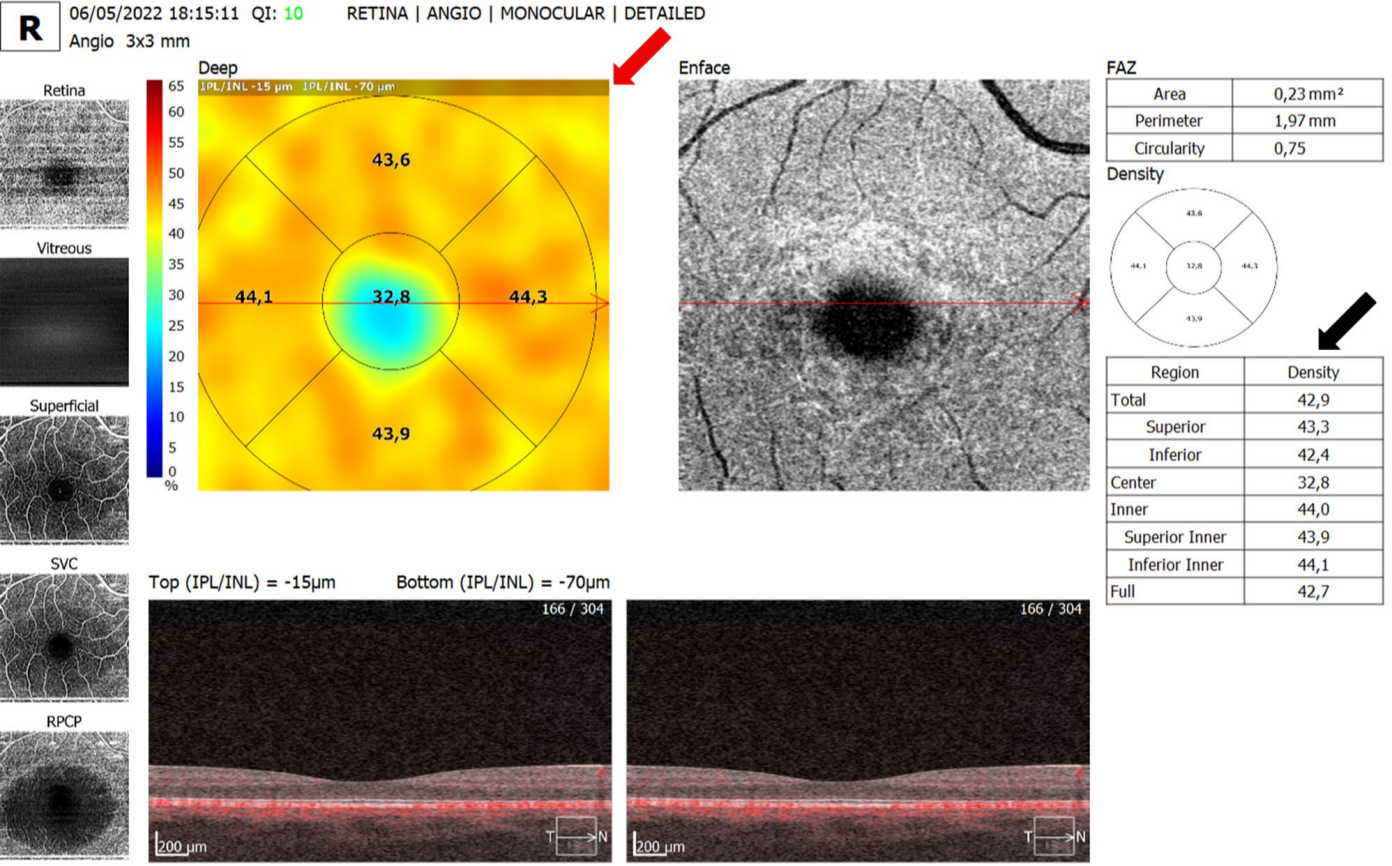
Deep capillary plexus vessel density map with quantitative OCTA parameters. Representative 3 × 3 mm OCTA deep plexus density map (red arrow) with corresponding quantitative metrics (black arrow). Default DCP segmentation applied. Scale bar = 200 μm; signal strength = 10/10

**Fig. 4:**
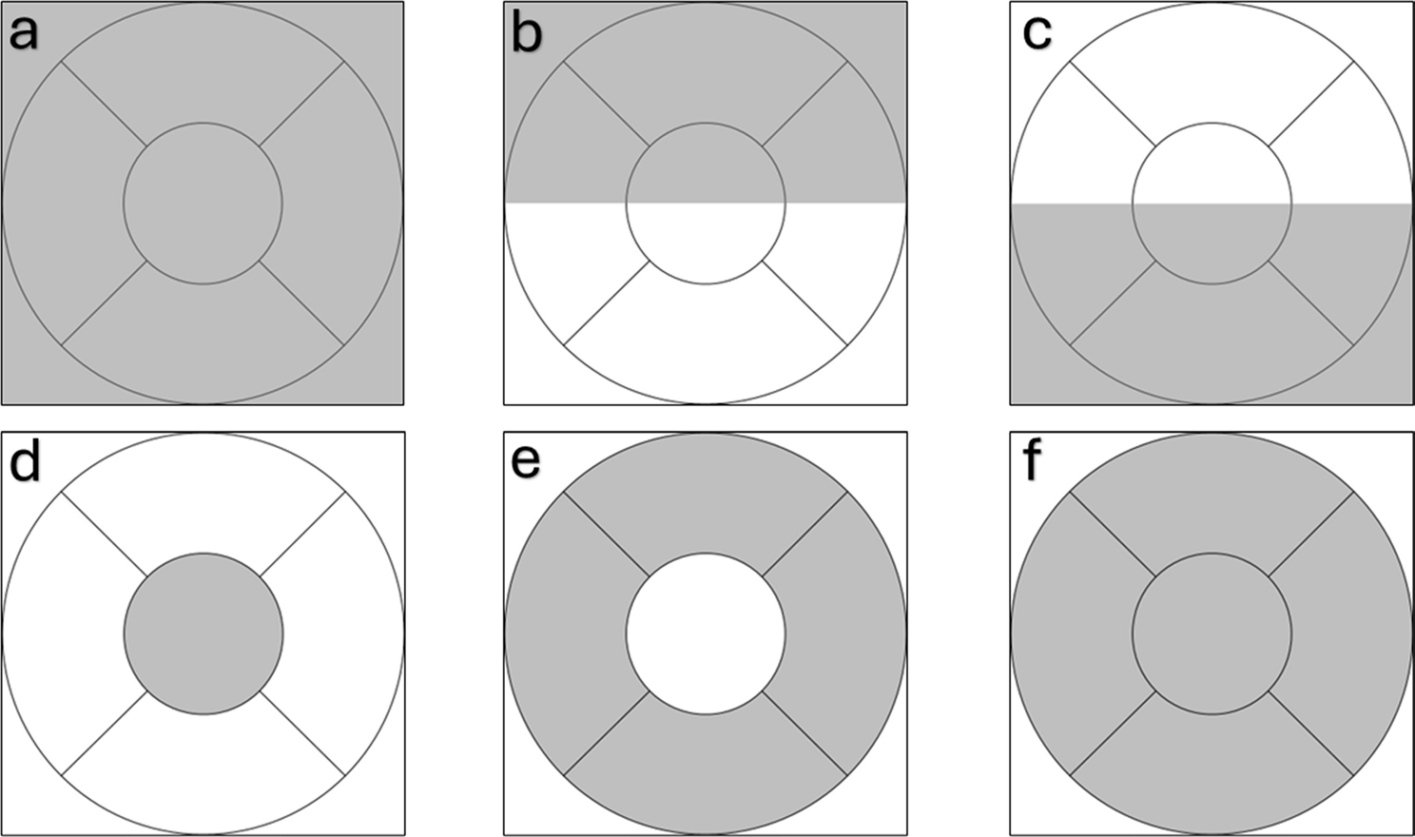
Schematic representation of the 3 × 3 mm macular scan. Gray regions indicate areas of vessel density measurement. Diagram illustrating the regions used for vessel density analysis in the 3 × 3 mm OCTA field: (**a**) total scan area; (**b**) superior; (**c**) inferior; (**d**) foveal (center); (**e**) parafoveal (inner ring); and (**f**) combined foveal and parafoveal region (full)

The ophthalmological variables assessed by OCTA were FAZ - area (mm²), perimeter (mm), and circularity index in the superficial capillary plexus (Fig. 2); superficial and deep capillary density (%) in the foveal (center), parafoveal (inner, superior, inferior, temporal, and nasal), fovea and parafovea (full) regions, and 3 × 3 mm field (total, superior, and inferior), as shown in Figs. 3 and 4. All OCT and OCTA measurements were calculated automatically, except for choroidal thickness, which was measured manually. Continuous outcomes are presented as mean ± SD with 95% confidence intervals.

### Statistical analysis

The prespecified primary outcomes were superficial and deep capillary plexus vessel density. Secondary outcomes included FAZ metrics, macular thickness, choroidal thickness, and exploratory subgroup analyses based on duration of contraceptive use.

All parameters were evaluated in both eyes of each participant. To avoid inter-eye correlation, eye laterality was randomized using a computer-generated number sequence, ensuring that each participant contributed one eye and that right and left eyes were distributed in a strictly balanced manner (50% each) across all study groups.

For the comparison among three independent groups, data distribution within each group was first assessed using the Shapiro–Wilk test to evaluate the assumption of normality. Homogeneity of variances was then examined with Levene’s test. When both assumptions were satisfied, a one-way ANOVA was performed, followed by Tukey’s test for multiple comparisons. In the presence of unequal variances but normally distributed data, Welch’s ANOVA was applied, with post hoc analysis using the Games–Howell test. When at least one group did not meet the normality assumption, the Kruskal–Wallis test was employed, and pairwise comparisons were carried out using the Dwass–Steel–Critchlow–Fligner test. No correction for multiple hypothesis testing was applied, as this was an exploratory analysis designed to identify potential physiological patterns without increasing type II error risk. This approach is aligned with recommendations for exploratory ophthalmic research where strict adjustment may mask meaningful biological signals [6].

The sample size was determined considering the deep capillary plexus (DCP) vessel density (3 × 3 mm) as the primary endpoint. Assuming a clinically relevant difference of 1.0% point between groups, a one-way ANOVA with three groups (α = 0.05) indicated that 29 participants per group were required to achieve 80% power. Our final sample size (40 per group) provides an estimated power of approximately 92%. All statistical tests were two-tailed, and a P-value of < 0.05 was considered statistically significant. Data analyses were performed with Jamovi software (The jamovi project, version 2.3.28), an open-source statistical platform built on the R statistical environment.

## Results

A total of 120 patients were evaluated and equally distributed into three groups of 40 patients each: COC, POC, and control groups. Once the predetermined number of patients was reached in each group, no new patients were included.

Image quality was high across all examinations. For structural OCT, the quality index was 9 in six scans and 10 in all remaining scans. For OCTA, quality indices were 8 in three scans, 9 in five scans, and 10 in the remaining examinations. No scans required exclusion.

No significant differences were observed among the three groups in terms of age, BMI, and ophthalmological examination. The data are presented in Table 1.

**Table 1:** Demographics and clinical characteristics of the subjects

	COC group (*N* = 40)	POC group (*N* = 40)	Control group (*N* = 40)	*P* value*
Age (years)	30.8 ± 4.49	32.6 ± 4.75	31.0 ± 7.28	0.185
BMI (kg/m²)	25.6 ± 3.68	26.7 ± 5.04	25.1 ± 5.05	0.303
VA (LogMAR)	0.015 ± 0.04	0.003 ± 0.02	0.010 ± 0.03	0.152
IOP (mmHg)	11.6 ± 2.53	10.8 ± 1.62	11.7 ± 2.34	0.168
Spherical equivalente (D)	− 1.30 ± 1.76	− 1.02 ± 1.46	− 1.02 ± 1.48	0.736

Values presented as mean ± standard deviation. VA: visual acuity; BMI: body mass index; COC: combined oral contraceptive; D: diopters; IOP: intraocular pressure; POC: progestogen-only contraceptives. *global comparison (ANOVA/Kruskal–Wallis)

Ten different pharmaceutical presentations of contraceptives were observed in the COC group. Four different presentations were observed in the POC group. The compositions of the COC and POC are shown in Tables 2 and 3, respectively.

**Table 2:** Composition of combined oral contraceptives

Estrogen	Progestogen	*N*
Ethinylestradiol 0.02 mg	Levonorgestrel 0.1 mg	2
Ethinylestradiol 0.03 mg	Levonorgestrel 0.15 mg	7
Ethinylestradiol 0.02 mg	Gestodene 0.075 mg	3
Ethinylestradiol 0.03 mg	Gestodene 0.075 mg	2
Ethinylestradiol 0.02 mg	Desogestrel 0.15 mg	2
Ethinylestradiol 0.02 mg	Drospirenone 3 mg	3
Ethinylestradiol 0.03 mg	Drospirenone 3 mg	4
Ethinylestradiol 0.04 mg / 0.03 mg	Desogestrel 0.025 mg / 0.125 mg	1
Ethinylestradiol 0.035	Cyproterone acetate 2 mg	14
Estradiol valerate 2 mg / 3 mg	Dienogest 2 mg / 3 mg	2

**Table 3:** Composition progestogen-only oral contraceptives

Progestogen	*N*
Desogestrel 0.075 mg	31
Dienogest 2 mg	6
Drospirenone 4 mg	2
Noretisterone 0.35 mg	1

Primary outcomes and selected key secondary outcomes are presented in the main tables for clarity. The full set of OCT and OCTA parameters evaluated in this study is provided in the Supplementary Material.

Across all evaluated parameters, including FAZ metrics, SCP and DCP vessel density in both foveal and parafoveal regions, and macular and subfoveal choroidal thickness, no statistically significant differences were detected among the groups, as shown in Table 4.

**Table 4:** Comparison of FAZ parameters, vessel density, and macular and choroidal thickness between three groups

	Parameter	COC group (*N* = 40)	POC group (*N* = 40)	Control group (*N* = 40)	*P* value*
FAZ	Area (mm²)	0.31 ± 0.10	0.31 ± 0.11	0.31 ± 0.14	0.960
VD-SCP (%)	Total (3 × 3 mm)	38.7 ± 3.20	38.7 ± 2.16	38.9 ± 1.69	0.661
	Superior	39.6 ± 2.72	39.5 ± 2.58	39.9 ± 1.43	0.884
	Inferior	37.6 ± 3.80	37.8 ± 2.67	38.1 ± 2.33	0.919
	Fovea (center)	18.3 ± 4.19	17.6 ± 3.76	18.2 ± 5.54	0.808
	Parafovea (inner)	40.0 ± 3.64	40.3 ± 2.38	40.4 ± 1.47	0.666
	Fovea + parafovea (full)	37.5 ± 3.67	37.7 ± 2.27	38.0 ± 1.51	0.789
VD-DCP (%)	Total (3 × 3 mm)	42.5 ± 1.36	42.5 ± 1.33	42.7 ± 1.47	0.307
	Superior	43.1 ± 1.17	42.7 ± 1.90	43.0 ± 1.03	0.495
	Inferior	42.0 ± 1.63	42.0 ± 1.53	42.1 ± 2.30	0.298
	Fovea (center)	31.6 ± 4.46	31.8 ± 3.10	31.8 ± 5.09	0.879
	Parafovea (inner)	43.7 ± 1.34	43.5 ± 1.74	43.7 ± 1.18	0.839
	Fovea + parafovea (full)	42.3 ± 1.60	42.18 ± 1.75	42.3 ± 1.45	0.563
Thickness (µm)	Fovea	224.5 ± 19.0	225.1 ± 17.8	227.3 ± 20.4	0.790
	Choroidal	337.1 ± 43.0	356.7 ± 55.9	329.1 ± 57.2	0.057

Values presented as mean ± standard deviation. COC: combined oral contraceptive; DCP: deep capillary plexus; FAZ: foveal avascular zone; POC: progestogen-only contraceptives; SCP: superficial capillary plexus; VD: vessel density. *global comparison (ANOVA/Kruskal–Wallis)

The influence of COC use duration on the analyzed parameters was also assessed by stratifying participants according to the median exposure time (≤5 years and >5 years). For FAZ metrics and vessel density in both the superficial and deep capillary plexuses, no differences were identified when comparing users with longer versus shorter duration of COC use or when comparing either subgroup with controls. Similarly, macular and subfoveal choroidal thickness did not differ among the groups. The main numerical findings are summarized in Table 5.

**Table 5:** Comparison of FAZ, vessel density, and macular-choroidal thickness between long- and short-term COC users, and controls

	Parameter	COC ≤ 5 years (*N* = 21)	COC > 5 years (*N* = 19)	Control group (*N* = 40)	*P* value*
FAZ	Area (mm²)	0.31 ± 0.10	0.31 ± 0.12	0.31 ± 0.14	0.999
VD-SCP (%)	Total (3 × 3 mm)	38.6 ± 2.79	38.8 ± 3.67	38.9 ± 1.69	0.495
	Superior	39.5 ± 2.63	39.7 ± 2.88	39.9 ± 1.43	0.726
	Inferior	37.6 ± 3.11	37.7 ± 4.53	38.1 ± 2.33	0.760
	Fovea (center)	17.9 ± 4.43	18.6 ± 3.99	18.2 ± 5.54	0.900
	Parafovea (inner)	39.9 ± 3.16	40.0 ± 4.20	40.4 ± 1.47	0.499
	Fovea + parafovea (full)	37.4 ± 3.18	37.5 ± 4.24	38.0 ± 1.51	0.664
VD-DCP (%)	Total (3 × 3 mm)	42.6 ± 1.03	42.5 ± 1.68	42.7 ± 1.47	0.349
	Superior	43.1 ± 0.94	43.0 ± 1.40	43.0 ± 1.03	0.947
	Inferior	42.0 ± 1.30	42.0 ± 1.96	42.1 ± 2.30	0.309
	Fovea (center)	31.8 ± 4.10	31.5 ± 4.94	31.8 ± 5.09	0.981
	Parafovea (inner)	43.7 ± 0.93	43.6 ± 1.71	43.7 ± 1.18	0.936
	Fovea + parafovea (full)	42.4 ± 1.18	42.3 ± 2.00	42.3 ± 1.45	0.973
Thickness (µm)	Fovea	222.7 ± 15.9	226.4 ± 22.2	227.3 ± 20.4	0.690
	Choroidal	330.4 ± 39.9	344.5 ± 46.2	329.1 ± 57.2	0.535

Values presented as mean ± standard deviation. COC: combined oral contraceptive; DCP: deep capillary plexus; FAZ: foveal avascular zone; SCP: superficial capillary plexus; VD: vessel density. *global comparison (ANOVA/Kruskal–Wallis)

The influence of POC use duration on the evaluated parameters was also assessed by stratifying participants according to the median exposure time (2 years). For FAZ metrics, vessel density in the deep capillary plexus, and macular and subfoveal choroidal thickness, no differences were identified among the three groups. The only statistically significant finding was a reduction in vessel density in the nasal parafoveal sector of the SCP in POC users with less than two years of use compared with controls (*P* = 0.033; post hoc *P* = 0.049), with a mean difference of 0.70 (95% CI, 0.03 to 1.37). This was the only significant between-group difference observed in the POC analysis and did not extend to any other OCTA or OCT parameter. Numerical estimates for this secondary finding are provided in the Supplementary Tables; the primary outcomes are summarized in Table 6.

**Table 6:** FAZ parameters, vessel density, and macula and choroidal thickness, between < 2 years and > 2 years and control groups

	Parameter	POC ≤ 2 years (*N* = 25)	POC > 2 years (*N* = 15)	Control group (*N* = 40)	*P* value*
FAZ	Area (mm²)	0.34 ± 0.12	0.27 ± 0.08	0.31 ± 0.15	0.274
VD-SCP (%)	Total (3 × 3 mm)	39.1 ± 1.57	38.2 ± 2.87	38.9 ± 1.69	0.311
	Superior	39.3 ± 2.64	39.8 ± 2.53	39.9 ± 1.43	0.733
	Inferior	38.0 ± 2.52	37.4 ± 2.96	38.1 ± 2.33	0.823
	Fovea (center)	17.5 ± 3.38	17.9 ± 4.45	18.2 ± 5.54	0.856
	Parafovea (inner)	40.4 ± 2.17	40.1 ± 2.77	40.4 ± 1.47	0.780
	Fovea + parafovea (full)	37.8 ± 1.95	37.5 ± 2.80	38.0 ± 1.51	0.734
VD-DCP (%)	Total (3 × 3 mm)	42.5 ± 1.47	42.6 ± 1.08	42.7 ± 1.47	0.356
	Superior	42.4 ± 2.22	43.2 ± 1.10	43.0 ± 1.03	0.074
	Inferior	41.8 ± 1.74	42.3 ± 1.07	42.1 ± 2.30	0.415
	Fovea (center)	31.3 ± 3.12	32.5 ± 3.04	31.8 ± 5.09	0.365
	Parafovea (inner)	43.3 ± 2.04	43.8 ± 1.09	43.7 ± 1.18	0.564
	Fovea + parafovea (full)	41.9 ± 2.05	42.4 ± 1.05	42.3 ± 1.45	0.578
Thickness (µm)	Fovea	221.1 ± 19.6	231.7 ± 12.3	227.3 ± 20.4	0.207
	Choroidal	349.6 ± 47.6	368.5 ± 67.6	329.1 ± 57.2	0.058

Values presented as mean ± standard deviation. DCP: deep capillary plexus; FAZ: foveal avascular zone; POC: progestogen-only contraceptives; SCP: superficial capillary plexus; VD: vessel density. *global comparison (ANOVA/Kruskal–Wallis)

## Discussion

Hormonal oral contraceptives are among the most widely used drugs worldwide. They are prescribed not only for contraception but also for managing several gynecological conditions, including polycystic ovary syndrome, endometriosis, dysmenorrhea, and abnormal uterine bleeding [7]. Despite their widespread use, significant gaps in knowledge remain regarding their systemic effects, particularly their influence on microcirculation.

Estrogen and progesterone receptors are expressed in multiple ocular structures, including the retinal pigment epithelium, neural retina, and choroid, suggesting a potential hormonal modulation of ocular vascular homeostasis [8]. Estrogen exerts vasodilatory, anti-inflammatory, and antioxidant effects through nitric oxide–mediated pathways, whereas the vascular effects of progesterone vary according to dose and progestin class, with some formulations associated with increased vascular resistance [9, 10]. These mechanisms provide biological plausibility for the hypothesis that exogenous hormones could influence retinal microcirculation, although this was not observed in the present study. In this context, it is essential to understand the impact of hormonal contraceptives on the retina, which is one of the few structures where microcirculation can be assessed in vivo in a noninvasive manner.

The impact of hormonal oral contraceptive use on different body systems remains under investigation. In the ophthalmological field, COC have been linked to several ocular pathologies. However, evidence shows that only retinal vascular lesions present a significantly increased risk, with a two-fold higher incidence among users [2]. These events include central retinal vein occlusion, retinal artery occlusion, and cerebral venous sinus thrombosis with ocular manifestations, and appear to be more frequent with older, higher-dose formulations [11]. The proposed mechanisms involve alterations in coagulation factors, anticoagulant proteins, and fibrinolytic activity, resulting in a prothrombotic state dependent on the estrogen dose and type of progestogen used. In contrast, progestogen-only contraceptives are considered safer with regard to thromboembolic and cardiovascular risks [12].

Several clinical reports support the association between COC use and retinal vascular pathology. Cases include central retinal vein occlusion in young women without other thromboembolic risk factors [13], acute macular neuroretinopathy in a 16-year-old [14], cerebral venous sinus thrombosis initially presenting with ocular symptoms in an 18-year-old user [15], papillophlebitis in a 20-year-old patient [16], and branch retinal artery occlusion in a 15-year-old [17]. Importantly, even with the lower estrogen doses in modern formulations, oral contraceptives continue to represent a relevant risk factor for ocular thrombotic events [18].

Recent studies have shown that vascular changes may occur in response to the use of exogenous hormones, including changes in retinal microcirculation, such as thickening of the nerve fiber layer and reduced capillary density [4, 5]. These changes may represent early signs of broader vascular complications, given the role of the retina as an accessible window for assessing the systemic microvasculature. Therefore, understanding the effects of hormonal contraceptives on retinal circulation is crucial for identifying possible risks associated with prolonged use, especially in populations at risk of vascular diseases.

In the present study, OCTA was used to assess whether the use of COC and POC would cause changes in retinal microvascularization when compared with individuals who did not use these medications. The great innovation of this study was to include, for the first time, a group of POC users, since the available literature had previously been limited to the analysis of COC [4, 5].

The robust sample of 120 patients allowed for a statistically reliable analysis of the data. Users were evenly distributed between three groups of 40 patients each and carefully selected to exclude possible confounding factors such as systemic and ocular diseases. This is the largest sample size of individuals studied in the reviewed studies. Two recent studies evaluated groups of 32 and 30 patients [4, 5].

The groups in the present study were homogeneous in terms of baseline variables such as age and BMI. This comparable demographic profile is relevant given that the literature indicates that such factors can influence OCTA parameters, although their effects are typically more evident in older populations or in individuals with extreme weight variations [19, 20]. Therefore, the observed homogeneity supports the premise that the lack of significant differences in the most of the evaluated parameters effectively reflects the absence of a clinically relevant effect of hormonal contraceptives on the ocular microvasculature in this cohort.

No differences were observed among the groups when evaluating the parameters obtained using OCTA. To date, only one study has compared OCTA parameters in patients using COC and those not using hormonal contraceptives. Icoz et al. (2023) [4] observed a reduction in deep vessel density in all regions studied in the group using COC. No differences were observed in the superficial vessel density or FAZ parameters, similar to the findings of the present study.

Analysis of OCT parameters revealed no significant differences in choroidal or macular thickness among the control, COC, and POC groups. Madendag et al. (2017) [21] compared macular and choroidal thickness in patients using COC and observed no difference in choroidal thickness between the COC group and the control group. The same authors found a statistically significant difference in the macular thickness in all parafoveal and perifoveal regions. Shaaban and Badran (2019) [3] reported a significant decrease in choroidal thickness and all macular regions in patients using COC.

The impact of COC use duration on retinal vascularization was assessed within the sample. As the median duration of COC use was 5 years, patients with >5 years of use were compared with patients with <5 years of use, and with the control group. No differences were observed between these subgroups, demonstrating that prolonged COC use does not alter retinal vascularization or thickness. No previous studies have made such comparisons. The studies conducted so far have not mentioned the duration of contraceptive use, simply stating that the use should be for a minimum period of 1 year.

Similarly, the duration of POC use was also evaluated. Unlike COC, the median duration of use was 2 years. The duration of use had no impact (difference) on FAZ parameters or vessel density in the deep capillary plexus. A marginally significant reduction in vessel density of the nasal parafoveal sector of the superficial capillary plexus was found in the POC subgroup with less than 2 years of use compared with controls. The clinical significance of this finding remains uncertain. These results are particularly relevant because previous studies did not stratify their samples according to the duration of use.

The lack of differences between shorter and longer durations of use suggests that, within the constraints of a cross-sectional design, prolonged hormonal contraceptive exposure was not associated with detectable cumulative changes in the retinal microvasculature. However, this interpretation should be made with caution, especially given the absence of longitudinal data.

Beyond the potential effects of hormonal exposure, OCTA has proven capable of detecting early microvascular alterations associated with other systemic medications, such as hydroxychloroquine and tamoxifen — drugs known to exert retinal toxicity [22–24]. These precedents demonstrate that subtle, drug-related retinal perfusion changes can be measurable in vivo, supporting the rationale for evaluating retinal microvasculature in hormonal contraceptive users.

Compared with systemic conditions well known to impair retinal microcirculation — such as diabetes, hypertension, and chronic smoking — hormonal contraceptives showed no comparable pattern of microvascular dysfunction in this cohort. OCTA studies have demonstrated marked reductions in vessel density and FAZ enlargement in early diabetes [25, 26], measurable capillary alterations in treated hypertension [27–29], and reduced perfusion among chronic smokers [30, 31]. In contrast, retinal parameters in COC and POC users were essentially indistinguishable from controls, supporting the notion that, in healthy women, hormonal contraceptives exert a substantially milder vascular influence than these systemic conditions.

Our findings of no significant microvascular alterations align with the established safety profile of hormonal contraceptives and contrast with the documented effects of other medications and systemic conditions on retinal vasculature. Unlike well-known retinal toxins, such as hydroxychloroquine and tamoxifen, which can induce measurable changes in vessel density and structure, our data did not reveal patterns suggestive of similar vascular effects in COC or POC users. This distinction is crucial, as it places these contraceptives in a category of low ocular risk. Moreover, the absence of an effect is further reinforced when compared with the subclinical vascular changes often associated with chronic systemic conditions like hypertension, diabetes, and smoking. The absence of any association, even with prolonged use, supports the absence of measurable microvascular differences in this cross-sectional sample; however, long-term ocular safety cannot be inferred from this study design.

This study had some limitations that should be considered when interpreting the results. First, given its cross-sectional design, causality cannot be inferred, and longitudinal studies are required to determine temporal and cumulative effects. Although contraceptive formulations were documented, subgroup analyses by estrogen dose or progestin class were limited by small numbers of users within each formulation. Future studies with larger stratified cohorts are warranted. We acknowledge that some physiological covariates were not assessed; nevertheless, this approach is common in OCTA studies of healthy subjects and remains appropriate for an exploratory design.

Given these limitations, future studies may contribute to a better understanding of the relationship between hormonal contraceptives and retinal circulation. Prospective studies with longitudinal follow-up could clarify whether vascular changes may occur or worsen with increased duration of use, or whether they are reversible after discontinuation.

## Conclusions

In this cross-sectional study, hormonal contraceptive use was not associated with meaningful differences in FAZ parameters, macular or choroidal thickness, or overall retinal vessel density. A small reduction in nasal parafoveal vessel density was observed among women using progestin-only contraceptives for less than two years compared with controls. However, the clinical relevance of this exploratory finding remains uncertain and requires confirmation in longitudinal studies.

## Supplementary Information

Below is the link to the electronic supplementary material.

Supplementary Material 1

## Data Availability

The datasets used and/or analyzed during the current study are available from the corresponding author on reasonable request.
